# Effects of School Nurse-Led Interventions in Collaboration with Kinesiologists in Promoting Physical Activity and Reducing Sedentary Behaviors in Children and Adolescents: A Systematic Review

**DOI:** 10.3390/healthcare11111567

**Published:** 2023-05-26

**Authors:** Yari Longobucco, Matteo Ricci, Susan Scrimaglia, Claudia Camedda, Laura Dallolio, Alice Masini

**Affiliations:** 1 Department of Health Sciences, University of Florence, 50134 Firenze, Italy; yari.longobucco@unifi.it; 2 Department of Biomedical and Neuromotor Sciences, Alma Mater Studiorum, University of Bologna, 40126 Bologna, Italy; matteo.ricci18@studio.unibo.it (M.R.); laura.dallolio@unibo.it (L.D.); alice.masini7@unibo.it (A.M.); 3 IRCCS Azienda Ospedaliero-Universitaria di Bologna, 40138 Bologna, Italy; claudia.camedda2@unibo.it; 4 Department of Medical and Surgical Sciences, Alma Mater Studiorum, University of Bologna, 40126 Bologna, Italy

**Keywords:** lifestyles, kinesiologist, public health, nurse, health promotion, school nurse, family and community nurse

## Abstract

The World Health Organization (WHO) recommends that schools adopt a whole-school strategy for healthy behaviors involving different health professionals. The present systematic review aimed to evaluate the efficacy of nurse-led interventions in collaboration with kinesiologists on physical activity and lifestyle behaviors’ outcomes in school settings. The protocol was registered in PROSPERO (ID: CRD42022343410). The primary research study was developed through the PICOS question: children and adolescence 6–18 years (P); school nurse-led interventions in promoting physical activity (PA) and reducing sedentary behaviors (I); usual lessons, no intervention focusing on PA (C); PA levels, sedentary behaviors, and healthy lifestyle behaviors (O); experimental or observational study with original primary data and full-text studies written in English (S). Seven studies were included. Interventions were heterogeneous: besides physical activities carried out in all studies, the interventions were based on different health models and strategies (counselling, face-to-face motivation, education). Five out of seven articles investigated PA levels or their related behaviors using questionnaires, and two used ActiGraph accelerometers. Lifestyle behaviors were assessed with heterogeneous methods. Five out of seven articles showed an improvement in at least one outcome after the interventions, whereas two papers showed a statistically non-significant improvement. In conclusion, school interventions involving nurses, also in association with other professionals such as kinesiologists, can be effective in reducing sedentary behaviors and improving healthy lifestyles in children and adolescents.

## 1. Introduction

The study of healthy behaviors among schoolchildren and adolescents has been an ongoing concern for more than 20 years [1]. This specific population has been the focus of public health promotion since statistics from several sources indicate that high-risk health behaviors are increasing. Not engaging in enough physical activities or not eating a balanced diet are examples of risk behaviors that are associated with chronic degenerative diseases and other adverse health outcomes in the future, including heart disease, stroke, and metabolic illnesses [2]. On the other hand, healthy habits, such as limiting sedentary behaviors and being active, are essential to youth development, wellbeing, and health [3]. Schools offer a special opportunity to encourage positive habits; for instance, the majority of US students spend 6 to 7 h per day, or a significant portion of their waking hours, at school [4]. The school setting provides extended access to a high percentage of kids and youth and a chance to lower chronic disease rates among the general population [4]. Internationally, several governments and health organizations advise schools to have practices and policies that promote the development of a learning environment where students are encouraged to make healthy decisions [4]. The World Health Organization (WHO), for instance, suggests that schools adopt a whole-school strategy for healthy behaviors with tactics that focus on the curriculum (i.e., learning, teaching, professional development), environment (i.e., physical, culture, policies, procedures), and partnerships (i.e., students, families, staff, community) [4]. Among all, sedentary behaviors have an impact on children’s and adolescents’ health (such as poor physical fitness and cardiometabolic health, reduced sleep duration, and pro-social behavior), but they also have long-term effects as they are associated with an increase in cardiovascular risk factors in young adulthood, including adiposity and metabolic syndrome [5]. For these reasons, for children and adolescents aged 5 to 17 years, the World Health Organization (WHO) recommends engaging in at least 60 min per day of moderate to vigorous physical activity (MVPA) and limiting the amount of time spent being sedentary [6]. However, according to the most recent data from a pooled study of 298 population-based surveys with 1.6 million participants, 81% of school-age adolescents worldwide in 2016 did not satisfy the PA recommendations [6]. In order to achieve these recommendations, many interventions aimed at promoting physical activities and limiting sedentary behaviors in children and adolescents have been studied, also with different professionals involved in cooperation with kinesiologists [7]. However, questions have been raised in recent years as to whether the intervention models used to date were adequate, with a proposal to shift from a risk-reduction, prevention-oriented approach to a person-based, development-oriented approach; this last paradigm involves a holistic approach [2]. Given the many professionals involved in the health promoting school movement, this latter aspect can be difficult. Building such group capacity through fostering social interaction, cohesion, participation, and political action is considered a competence of nurses [8]. Consequently, to the acknowledgement that this figure received during the COVID-19 pandemic, the family and community nurse (FCN) has recognized skills in this specific area, as defined by the ENhANCE (EuropeaN-curriculum-for-fAmily-aNd-Community-nursE) curriculum of FCN’s core competencies [9,10].

To the best of our knowledge, the evidence of the effects of the involvement of nurses in this setting has never been pooled together. For this reason, the present systematic review can cover a gray area of research that needs to be further explored, mainly due to the relevance of new professionals’ figures (e.g., school nurses and kinesiologists) as important health ambassadors. The aim of this work was to evaluate the efficacy of nurse-led interventions on physical activities’ and lifestyle behaviors’ outcomes in the school setting. The review question was: what is the existing evidence about the effects of nurse-led interventions carried out in a school context in collaboration with kinesiologists or similar on physical activity levels and sedentary and lifestyle behaviors in children and adolescents?

Due to the rise of sedentary behaviors and physical inactivity in young people and the crucial role of school nursing in promoting health, it is relevant to provide scientific knowledge to contribute to nursing science and its role in contrasting these trends. Nevertheless, the recent widespread dissemination of FCN in some European countries, consequently due to the COVID-19 pandemic [10], presents a valuable opportunity for the implementation of this professional figure in this setting, and this review aimed to help build a starting point in this area.

## 2. Materials and Methods

### 2.1. Study Design, Search Strategy, and Selection Criteria

A systematic review of published studies reporting the effects of school nurse-led intervention in promoting physical activity and reducing sedentary behavior in children and adolescents was performed in accordance with the preferred reporting items for systematic review and meta-analyses (PRISMA) statement [11].

The protocol was registered in the International Prospective Register of Systematic Reviews (ID: CRD42022343410). The primary research study was developed through the PICOS question (population, interventions, comparators, outcome, study design) using the following research items: school children and school adolescence 6–18 years (P); school nurse-led interventions in collaboration with kinesiologists in promoting physical activity and reducing sedentary behaviors (I); usual lessons, no intervention focusing on physical activity (C); physical activity levels, sedentary behaviors, and healthy lifestyle behaviors (O); experimental or observational study with original primary data and full-text studies written in English (S).

The above inclusion and exclusion criteria were decided based on the need to investigate nurse-led interventions in primary and secondary schools with the goal of promoting correct lifestyles and preventing chronic and metabolic conditions. Preschool children from 3 to 6 years old were excluded, as school nurses and kinesiologists are not present in kindergarten settings. All multicomponent approaches focused only on healthy diet and sleep hygiene with no physical activity or sedentary behavior component were excluded since our aim was to evaluate school nurse-led interventions in collaboration with kinesiologists. Finally, studies performed in other settings, such as hospitals, community centers, and sports associations, were excluded.

All types of studies were included due to the nature of these interventions, which are very complicated to conduct following only a randomized control design. In addition, other study designs may have been used to describe existing experiences (i.e., observational studies). Studies with no original data were excluded.

Medline (Pubmed), CINAHL (EBSCO), Cochrane Central Register of Controlled Trials (CENTRAL), SPORTdiscus (EBSO) were the electronic databases that were interrogated for researching all the studies published before the 11th of March 2022. These databases represented the gold standard for conducting a systematic review.

The review was restricted to studies in English with a publication date limit of 10 years, as they were focused on recent interventions. This criterion was applied considering that in Europe, school nurses are emerging professional figures. Based on the PICO, search terms were created using the following keywords and Boolean terms in order to be as sensitive and specific as possible: ((“School Nurse” OR “School Nurses” OR Nursin* School OR School Nursin*) OR “community health nursing” OR Public Health Nurs* OR “physical education teacher”) AND (Physical Education, Training OR Physical Education OR Exercis* OR Physical Activity OR Activit* Physical OR Physical Activities OR Exercis* Physical OR Physical Exercis* OR Acute Exercis* OR Exercis* Acute OR Exercis* Isometric OR Isometric Exercis* OR Exercis* Aerobic OR Aerobic Exercis* OR Exercise Trainin* OR Trainin* Exercise) AND (Child OR Children) AND (School OR Schools OR Primary Schools OR Primary School OR School, Primary OR Schools Primary OR Schools Secondary OR School Secondary OR Secondary School OR Secondary Schools OR Behavior Sedentary OR Sedentary Behaviors OR Sedentary Lifestyle OR Lifestyle Sedentary OR Physical Inactivity OR Inactivity Physical OR Lack of Physical Activity OR Sedentary Time OR Sedentary Times OR Time Sedentary). When necessary, the search string was adapted to perfectly fit in each database. Criteria for inclusion applied in the systematic review were the following: (1) population that included children and adolescents, regardless of gender and ethnicity, 6–18 years of age attending primary, secondary, and high school; (2) nurse-led interventions also in collaboration with physical education teachers that promote physical activity intervention, reduce sedentary behaviors, and improve healthy lifestyle behaviors; (3) studies with or without a control group in which participants did not receive any intervention or received interventions that were not based on school nursing; (4) experimental or observational studies with original primary data and full-text studies written in English; (5) studies with interventions involving school settings regardless of the country in which the school is located; (6) studies with physical activity levels, sedentary behaviors, and lifestyle-related behaviors as outcomes. Criteria for exclusion were the following: (1) preschool children 3–6 years of age, adults, and workers; (2) studies focusing exclusively on multicomponent interventions based on healthy nutrition and sleep hygiene; (3) study protocols or other papers without original data. The PICO inclusion/exclusion criteria are described in Table 1.

### 2.2. Data Extraction

YL and AM independently performed the literature research. Studies potentially eligible according to the inclusion criteria, after removal of duplicated studies, were identified from the title, abstract, and/or portions of the text by LD, YL, CC, AM, and SS; full texts of relevant studies were extracted and assessed independently by six reviewers (LD, YL, CC, AM, SS, and MR) according to the PICOS question (Table 1). Inconsistency regarding the eligibility of the studies was resolved through discussion and consensus between reviewers.

Data were extracted by the authors following the standardized rules for study collection. The details were collected in a standardized paper form, based on the method provided by the Cochrane Reviewers’ Handbook [12], that included: DOI, first author’s name, year of publication, title, type of study, presence of a control group, enrollment period, country, setting, study population with ages and number of participants in both experimental (EG) and control (CG) groups, percentage of males, type, frequency, and duration of the interventions, primary and secondary outcomes, and method/questionnaires/test used to measure the outcomes and results. Results were described as mean ± SD where possible. Divergences were solved again by consensus between the authors.

Unclear information or additional information regarding the interventions was requested by contacting the corresponding authors. In this specific review, all the authors responded, and no papers were excluded for this reason.

### 2.3. Quality Assessment and Risk Bias

A risk of bias critical appraisal of each article included in the review was conducted independently by all six authors (LD, YL, CC, AM, SS, MR), using the “Risk of Bias in Non-Randomized Studies- of Interventions” tool (ROBINS-I) [13] and the “Revised Cochrane risk-of-bias tool for randomized trials (RTC)” (ROB2) [14]. Conflicts over the quality scores were resolved through discussion and consensus among the reviewers.

The “Risk of Bias in Non-Randomized Studies- of Interventions” tool covers seven domains: (1) bias due to confounding; (2) bias in selection of participants into the study; (3) bias in classification of interventions; (4) bias due to deviations from intended interventions; (5) bias due to missing data; (6) bias in measurement of outcome; and (7) bias in selection of the reported results. The response options for each domain level were the following: low, moderate, serious, or critical risk of bias, and no information. The “Revised Cochrane risk-of-bias tool for RCTs presents five categories of domain: (1) risk of bias arising from the randomization process; (2) risk of bias due to deviation from the intended intervention (effects of assignment to intervention or effect of adhering to intervention); (3) missing out data; (4) risk of bias in measurement; and (5) risk of bias in selection of the reported results. These categories are translated as a high or low risk of bias or some concerns when the study is judged to raise some concerns in at least one domain for the result. All authors reviewed and approved the final manuscript.

## 3. Results

### 3.1. Search Results

**Figure 1:**
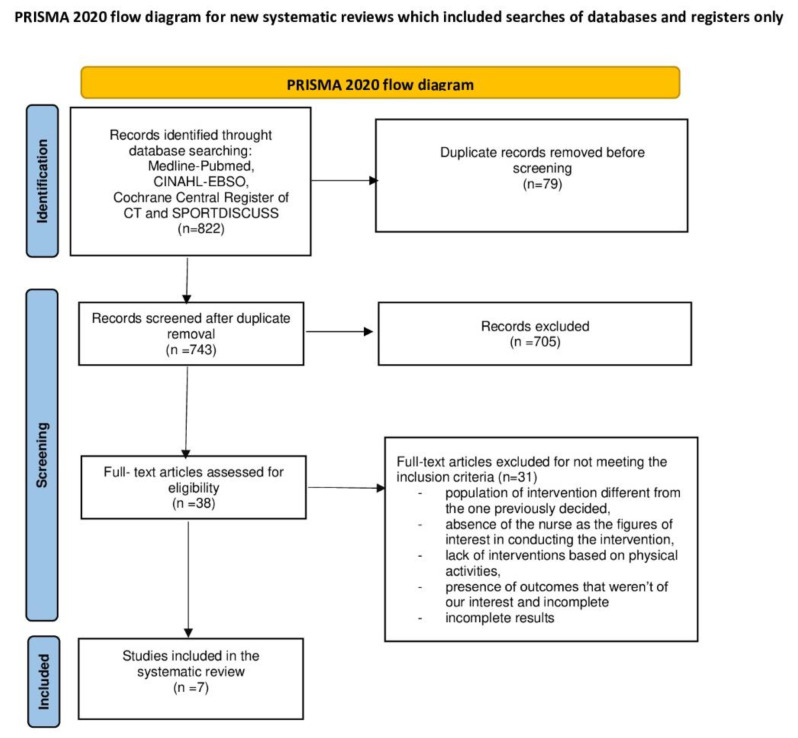
Prisma Flow diagram.

The number of potentially relevant articles identified through the literature research was 822. After excluding duplicates, 743 were screened, and 705 of them were removed based on title, abstract, and/or portions of the text. Finally, 38 papers were eligible for full-text reading, and 31 were later excluded. As shown in Figure 1, seven papers were included in this systematic review. Specifically, the main causes were the following: population of intervention different from the one previously decided; absence of the nurse as the figure of interest in conducting the intervention; lack of interventions based on physical activities managed by a kinesiologist or physical education teacher or trainer; presence of outcomes that were not of our interest; and incomplete results.

### 3.2. Study Characteristics and Data Extraction

The main data extracted from the seven included studies is presented in Table 2. As shown, the geographic origins of the articles were: USA (n = 4) [15,16,17,18], South Korea (n = 2) [19,20], and Turkey (n = 1) [21]. The study design and characteristics were heterogeneous. A total of five papers were RTC [15,17,18,20,21], and two [16,19] were quasi-experimental studies. The sample range varied from 1519 [15] to 69 [16]. Ages ranged from 8 [18,20] to 16 [17]. The length of the interventions varied from 6 [18] to 32 weeks [17], and the frequency varied from 30 [17] to 90 min of exercise [15,16,21] and from 15–20 [15,16] to 30 min of counseling [19]. The types of interventions were heterogenous: besides physical activities carried out in all papers and led by a kinesiologist, physical education teacher, or trainer [15,16,17,18,19,20,21], the interventions were based on different health promotion models and strategies, such as counseling sessions [15,16,17], face-to-face motivation with health professionals [15,16], nutrition and education modification [18], trans-theoretical model-based exercise counseling [19], health education groups in which warm-up games were played and questions and discussions were taken [21], and multilevel interventions conducted on different levels (children, parents, and center-based strategies) [20]. Starting from the RCTs, the aim of Robbins et al. [15] was to research successful interventions for increasing and sustaining girls’ moderate-to-vigorous physical activity (MVPA). The intervention was based on the health promotion model [22,23]. The outcomes were physical activity levels, measured with ActiGraph GT3X+ accelerometers, and BMI (kg/m^2^). No significant differences occurred between the intervention and control groups in the post-intervention MVPA. The aim of Whright et al. [18] was to evaluate the impact of a nurse-directed, school-based, coordinated, and family-centered lifestyle program on activity behavior and body mass index. The intervention used was the Kids N Fitness (KNF) intervention [24]. The primary outcomes were physical activity level and sedentary behavior, measured with the Child and Adolescent Trial for Cardiovascular Health (CATCH) School Physical Activity and Nutrition (SPAN) student questionnaires [25]. The intervention group showed an increase in daily activity and in physical exercise attendance, as well as a decrease in BMI and time spent in front of TV. Whereas Altunkurek et al. [21] focused on the effects of wellness coaching on both wellness and health behaviors in early adolescents. The adolescents were randomized into three groups: wellness coaching group, health education group, and control group. The outcomes were collected as follows: demographic information form and adolescent lifestyle scale (ALPS), five-factor wellness scale adolescent form (5F-Wellness-AF) [26], and BMI. Groups were compared in pairs. The wellness coaching group and the control groups’ mean 5F-Wellness-AF totals, as well as the one between the wellness coaching and health education groups’, were statistically significant. Regarding the ALP score, there was no difference between the health education and control groups’ means. The wellness coaching and control groups’ mean totals were significantly different. There was no significant difference between the wellness coaching and health education group means and between the health education and control group means. Pbert et al. [17] focused on the lookin’ good feelin’ good school-based program [27] for overweight and obese children. The main outcomes were physical activity levels, measured with ActiGraph Mode GT1M, sedentary behavior, described using the youth risk behavior survey [27], dietary intake and dietary behaviors, assessed by dietary recall interviews and an eight-item instrument [28,29], and BMI (kg/m^2^). Results showed that the intervention group did not differ in BMI compared to the control group; however, eating behaviors improved as breakfast was eaten on more days/week and the mean number of days of physical activity increased in the first group at follow-up. There were no statistical differences between the two groups.

Considering the last RCT examined, Choo et al. [20] analyzed the effects of a multilevel intervention program for obesity prevention among vulnerable children based on three levels (child [30,31], parent, and center). Children’s knowledge and healthy style behaviors were collected through questionnaires developed by the principal investigator and self-reports that assessed eating and activity behaviors. Obesity status was calculated using body weight and height, and additionally, BMI z-scores were also measured. The authors found that the intervention group showed significant improvement in knowledge and in total composite scores of healthy lifestyle behaviors compared to the control group, but not for obesity status or BMI z-scores, as no significant difference was found between groups.

Moving to the quasi-experimental studies, Ham et al. [19] carried out a study with the purpose of evaluating the effects of the Transtheoretical Model [32], based on after-school exercise counseling offered with music skipping rope exercise. The main outcomes were changes in stage of exercise behavior and BMI (kg/m^2^). Stages of change were individuated with questions developed by Marcus and Owen [33]. The results showed statistical differences in BMI between the control and intervention groups, as it increased in the first group but did not change in the second. No significant results were found regarding the advancing stages. Finally, Robbins et al. (2012) [16] conducted a pilot study based on the health promotion [22] model. Its intention was to determine whether girls receiving nurse counseling and an after-school physical activity club improved in physical activity, cardiovascular fitness (measured with an ActiGraph GT1M Accelerometer, ActiGraph LCC: Pensacole, FL, USA), BMI, percentage body fat, and waist circumference. Results did not show statistical significance, although the intervention group slightly improved in MVPA.

Different tools were used to measure the same outcomes in the papers analyzed. In three studies, PA was assessed using ActiGraph accelerometers [15,16,17]. Altunkurek et al. used the ALPS scale [21], which consisted of 44 items measured on a five-point Likert scale and divided into seven subscales (health responsibility, physical activity, nutrition, positive life perspective, interpersonal relationships, stress management, and spiritual health). Whereas Wright used the CATCH-SPAN questionnaire, which assessed activity behaviors utilizing five items (daily physical activity, participation in team sports, attendance at PE class, and TV computer/video game use) [18,25].

### 3.3. Quality Assessment and Risk of Bias Results

The risk of bias in the included studies was assessed using different tools, as explained in the Methods section. The risk of bias assessment was described in Table 3. Two of the included papers [16,19] had a quasi-experimental design and were assessed by the ROBINS-I scale. Robbins et al. obtained a low risk of bias, while Ham et al. were assessed as moderate due to the moderate risk of confounding bias and outcome measure bias. The ROB-2 scale was used to assess the RCT studies. In general, the risk of bias in the majority of the RCT studies included [17,20,21] was assessed as some concern, mainly due to the unclear procedures in the randomization process (Risk of bias arising from the randomization process) and the unblindness of participants (Risk of bias due to deviations from the intended interventions). The study conducted by Robbins et al. (2019) [15] was judged to have a high risk of bias due to deviations from the intended interventions. The risk of bias for the unblinded participants was the principal domain, causing a downgrade in the quality score. We are aware that this limitation derives from the nature of the experiment itself since the interventions consist of physical activity and counseling/education about correct lifestyles. This leads to the inability to create blind operators and participants.

## 4. Discussion

As stated above, the aim of the present paper was to evaluate the efficacy of nurse-led interventions on physical activity and lifestyle behaviors’ outcomes in the school setting. Finally, the present work includes and systematically analyzes seven articles. Five out of seven articles [17,18,19,20,21] investigated PA levels or their related behaviors using different questionnaires; only Robbins et al., in both of their papers [15,16], assessed this outcome with a more objective method, using ActiGraph accelerometers. Lifestyle behavior was assessed through different questionnaires, therefore, due to the heterogeneity of the instruments used, it was difficult to make a comparison. Only Pbert L. et al. [17] directly assessed dietary intake, while all papers, except for Robbins et al. (2019) [15], assessed body composition outcomes, although mainly using a coarse outcome such as BMI [34]. The risk of bias assessment gave highly variable results, ranging from low risk [16] to high risk [15]; most of the included articles raised some concerns. Five out of seven articles showed an improvement in at least one outcome after the intervention; only Robbins et al. [15,16], in both papers, obtained no differences after the intervention, although they showed a slight improvement. The possible reasons were different: the 2012s’ work was a pilot study, mainly focused on the feasibility of the intervention, while for the 2019s’ work, the participants’ attendance was less than optimal. Schoolchildren and adolescents were the main population of interest, as healthy habits are essential for their development, wellbeing, and health [3]. WHO estimates that more than 80% of schoolchildren and adolescents, at a global level, are insufficiently physically active [6,35]. Many interventions have been tested in the last decade [1], with different health professionals involved. This systematic review highlights the effectiveness of interventions involving nurses, in collaboration with other figures such as kinesiologists, in promoting health in school settings. This is consistent with the competencies of both figures, which complement each other. Nurses have competencies in health promotion [36,37]. Moreover, community nurses, specialized figures with an emerging role [9], have specific skills in this practice [38]; nevertheless, school nursing is a specific expertise of theirs [10]. In addition, school nursing is a cost-beneficial intervention for public health [39]. Moreover, it is well known that exercise and sport science related to health involve not only physicians and other licensed health care practitioners but other figures as well, such as the kinesiologist, exercise trainer, or physical education teacher [40]. Despite this, kinesiologists started to be systematically involved in health promotion in schools only in the last few years [41]. To the best of our knowledge, this is the first systematic review that synthesizes the available evidence on health promotion interventions in schools, especially those conducted with the integration of these two professionals. However, as can be guessed from the small number of articles found, those interventions are still not applied and evaluated enough. The main limitation of this work is the strong heterogeneity of the interventions adopted in the included papers. This aspect severely limits the generalizability of the results. Despite limited data, this systematic review suggests that nurses, in association with other figures such as kinesiologists, are important ambassadors in school health promotion; however, nurse-led interventions in schools are not sufficiently studied. Therefore, it is necessary to conduct further research on this topic.

## 5. Conclusions

Strategies for the reduction of sedentary behaviors and the improvement of healthy lifestyles are urgently needed for public health. Nowadays, multi-component and interdisciplinary interventions represent the most effective way to improve health-related behaviors. To achieve that, nurses can act as case managers for these health pathways. Moreover, as specific competences in physical education are needed, professionals such as kinesiologists should be involved in this type of treatment in collaboration with school nurse figures. Our review includes preliminary experiences that lead to an improvement in health behaviors’ outcomes. However, despite these results, further evidence in this field is needed.

## Figures and Tables

**Table 1:** Inclusion/exclusion criteria based on PICO.

Parameter	Inclusion Criteria	Exclusion Criteria
Population	Children and adolescence	Preschool children 3–6 years old
	Any gender and ethnicity	Adults
	6–18 years old	Workers
Intervention	Nurse-led interventions also in collaboration with kinesiologists that promote physical activity intervention, reduce sedentary behaviors, and improve healthy lifestyle behaviors. Interventions involving school setting	Studies focused on multicomponent intervention based only on healthy nutrition and sleep hygiene
Comparator	Studies with or without control group in which participants did not receive any intervention or received interventions that are not based on school nursing	
Outcome	Physical activity levels, sedentary behavior, lifestyle behaviors	Absence of physical activity levels
Study design	Experimental or observational studies with original primary data and full-text studies written in English	Study protocols or other papers without original data

**Table 2:** Data Extraction.

Author; Year; Country; Study Design	Study Population	Intervention	Outcomes	Results
Robbins L. B. et al. [15]. 2019; USA; RCT	n = 1519 (EG:753; CG:766) Males: n = 0 (0.00%) Age: EG:12.05 ± 0.99; CG:12.05 ± 1.02 Enrollment period: 2011–2016 Setting: Public Schools (5th–8th grades)	EG: The intervention was based on the Health Promotion Model and Self-Determination Theory and included three components: (i) an afterschool PA club at each school conducted by a club manager and three to four instructors; (ii) two face-to-face motivational, individually tailored counseling sessions (one at the beginning and other at the end of intervention) with a health professional having experience with adolescents (e.g., registered/school nurse); and (iii) an interactive Internet-basedsession via an iPad set up by the researchers at each school. CG: No intervention. Time: (i) 90 min of exercise; (ii) 15–20 min for each counseling session. Duration: 3 days/week (17 weeks).	*Primary Outcomes:* Physical Activity Levels (MVPA); BMI (kg/m^2^) *Methods/Questionnaire/Test:* ActiGraph GT3X+ accelerometers; BMI (kg/m^2^) *Other Outcomes:* Pubertal Stage (Pubertal Development Scale)	**T0 vs. T1 differences between EG and CG.** Physical Activity Levels: T0 EG: 3.03 min/h, T1 EG: 3.27 min/h; T0 CG: 2.92 min/h, T1 CG: 3.27 min/h; (*p* = n.s.). BMI: No information.
Wright K. et al. [18] 2012; USA; RCT	n = 251 (EG:121; CG:130) Males: n = 101 (40.24%) Age: EG:9.0 ± 1.6; CG:8.3 ± 1.1 Enrollment period: January 2008–September 2010 Setting: Primary Schools	EG: The intervention is conducted by registered nurses, trained community health workers and a physical education specialist. Sessions consisted of three components: physical activity, nutrition education/behavior modification, and family involvement. CG: The group participated in the standard physical activity program given by their respective schools and did not receive any physical or nutrition education. Time: 90 min. Duration: 6 weeks.	*Primary Outcomes:* Physical Activity Levels; Sedentary Behavior; BMI (kg/m^2^) *Methods/Questionnaire/Test:* CATCH SPAN Questionnaire (daily physical activity, attends PE class, TV viewing); BMI (kg/m^2^) *Other Outcomes:* Blood Pressure	**T0 vs. T1 differences between EG and CG.** Physical Activity Levels: T0 vs. T1 Increased participation in MVPA for male and female The effect was for both males (*p* = 0.002) and females (*p* = 0.005) at 12 months. T0 vs. T1 Increased participation in PE The effect was sustained for both males (*p* = 0.003) and females (*p* = 0.002). Sedentary Behavior: T0 vs. T1 Decreased TV viewing The effect was sustained at 12 months for males only (*p* = 0.030). BMI: In female students in the KNF group, BMI (*p* = 0.047) and BMI z-score (*p* = 0.05) decreased The effects were sustained for 12 months. While BMI and BMI z-scores decreased in males, this was not significant
Ham O. K. et al. [19] 2016; South Korea; Quasi-experimental study	n = 75 (EG: 48; CG: 27) Males: n = 43 (57.33%) Age: EG:10.77 ± 1.17; CG:10.26 ± 0.86 Enrollment period: April 2011–December 2011 Setting: Primary Schools	EG: eight-session individual counseling, 12 week music skipping rope exercise and booster counseling 3 months after the intervention. CG: one-session individual counseling, 12 week music skipping rope exercise. Time: (i) 30 min each TTM counseling (eight counseling sessions: four consecutive weeks for the first month and every other week for the last 2 months); (ii) 60 min of skipping rope exercise each week. Duration: 3 months.	*Primary Outcomes:* Stages of Change in Exercise Behavior; BMI (kg/m^2^) *Methods/Questionnaire/Test:* One question developed by Marcus and Owen aimed at the classification of the participant to one out of five categories; Electronic scales for measuring height and weight; Fourteen items of decisional balance developed by Marcus and Owen; BMI (kg/m^2^) *Other Outcomes:* Exercise; Self-Efficacy; Glucose Tolerance and Lipid Profile	**T0 vs. T1 differences between EG and CG.** Stages of Change in Exercise Behavior: 36.2% of EG and 17.4% CG advanced their exercise behavior by at least one stage; (*p* = n.s.). BMI: T0 EG 24.35 ± 2.73, T1 EG 24.37 ± 2.73; (*p* = 0.010) T0 CG 24.22 ± 2.24, T1 CG 24.99 ± 2.55; (*p* = n.s.).
Robbins L. B. et al. [16] 2012; USA; Quasi-experimental study	n = 69 (EG: 37; CG: 32) Males: n = 0 (0.00%) Age: EG:11.49 ± 0.67; CG:11.44 ± 0.84 Enrollment period: Spring 2009 Setting: Middle Schools	EG: (i) A 90 min after-school physical activity club offered at the middle school 5 days a week for 6 months (total of 98 sessions) and (ii) a face-to-face motivational, individually tailored counseling session with a registered (school) nurse during the school day every other month over the 6 months (total of three 20 min sessions were planned). The counseling sessions the girls had with the nurse occurred during the school day to capitalize on required school attendance. CG: (i) A 90 min after-school workshop once a month for 6 months (total of six workshops) and (ii) a face-to-face session with a registered (school) nurse during the school day every other month over the 6 months (total of three 20 min sessions were planned). Each workshop focused on one of the following health-promoting topics: (1) caring for my body; (2) fashion, hair, and nail tips; (3) sun and food safety; (4) healthy relationships and friendship; (5) building self-esteem; and (6) career exploration. The same workshop was offered on two consecutive days each month to enhance the opportunity for participation. Each session with the nurse included a discussion of two of the six topics. Time: (i) 90 min after-school physical activity club 5 days a week for 6 months (total of 98 sessions); (ii) face-to-face counseling sessions during the school day every other month over the 6 months (total of three 20 min sessions). Duration: 24 weeks.	*Primary Outcomes:* Physical Activity Levels (Minutes of MVPA/Hour); Cardiovascular Fitness (Progressive Aerobic Cardiovascular Endurance Run-PACER); BMI (kg/m^2^); Percentage Body Fat; Waist Circumference *Methods/Questionnaire/Test:* ActiGraph GT1M accelerometers; BMI (kg/m^2^) *Other Outcomes:* Perceived Benefits of and Barriers to Physical Activity; Perceived Physical Activity Self-Efficacy; Interpersonal Influences; Enjoyment of Physical Activity	**T0 vs. T1 differences between EG and CG.** Physical Activity Levels: T0 EG 0.69 ± 0.28, T1 EG 1.05 ± 0.41; T0 CG 1.08 ± 0.69, T1 CG 1.20 ± 0.65; (*p* = n.s.). Cardiovascular Fitness: T0 EG 10.50 ± 4.72, T1 EG 11.29 ± 7.28; T0 CG 14.10 ± 7.44, T1 CG 12.73 ± 7.54; (*p* = n.s.). BMI: T0 EG 25.94 ± 7.39, T1 EG 26.59 ± 7.40; T0 CG 24.88 ± 7.98, T1 CG 25.50 ± 7.90; (*p* = n.s.). Percentage Body Fat: T0 EG 34.02 ± 11.28, T1 EG 34.02 ± 10.83; T0 CG 31.98 ± 11.60, T1 CG 32.74 ± 11.25; (*p* = n.s.). Waist Circumference: T0 EG 80.78 ± 18.45, T1 EG 80.45 ± 17.17; T0 CG 79.57 ± 17.80, T1 CG 79.10 ± 15.58; (*p* = n.s.).
Altunkurek S. Z. et al. [21] 2019; Turkey; RCT	n = 132 (Wellness Coaching Program Group-WCPG: 33; Health Education Group-HEG: 33; Control Group-CG: 66) Males: n = 64 (48.5%) Age: 12–15 years old Enrollment period: September 2016–December 2016 Setting: 8th Grade Schools	**WCPG (Wellness Coaching Program Group):** Three main parts: physical activity, individual interviews, and group education. HEG (Health Education Group): A warm-up game was played in the first 5 min, and the last 10 min were devoted to questions and discussion. The content of the sessions was similar to the wellness coaching education. CG: No intervention. Time: (i) 90 min session 1 day a week of WCPG; (ii) 45–60 min once a week of HEG. Duration: 12 weeks.	*Primary Outcomes:* Five-Factor Wellness Scale-Adolescent Form (5F-Wellness-AF) Total Average; Adolescent Lifestyle Scale (ALPS) Total Average; BMI *Methods/Questionnaire/Test:* Five-Factor Wellness Scale-Adolescent Form (5F-Wellness-AF); Adolescent Lifestyle Scale (ALPS); Wellness Coach Individual Interview; Demographic Information Form; BMI *Other Outcomes:* ---	**T0 vs. T1 differences between EG and CG.** 5F-Wellness-AF Total Average: T0: WCPG < CG = 114.45 ± 16.55 < 128.33 ± 17.16; (*p* < 0.001). WCPG < HEG = 114.45 ± 16.55 < 125.73 ± 12.73; (*p* = 0.003). CG > HEG = 128.33 ± 17.16 > 125.73 ± 12.73; (*p* = n.s.). T1: WCPG < CG = 151.33 ± 6.02 > 126.82 ± 16.48; (*p* < 0.001). WCPG < HEG = 151.33 ± 6.02 > 129.03 ± 13.87; (*p* < 0.001): CG > HEG = 126.82 ± 16.48 < 129.03 ± 13.87; (*p* = n.s.). ALPS total average: T0: WCPG < CG = 112.88 ± 16.79 < 123.39 ± 18.70; (*p* = 0.021) WCPG < HEG = 112.88 ± 16.79 < 122.76 ± 17.49; (*p* = n.s.). CG > HEG = 123.39 ± 18.70 > 122.76 ± 17.49; (*p* = n.s.). T1: WCPG < CG = 158.82 ± 8.20 > 125.50 ± 18.62; (*p* < 0.001). WCPG < HEG = 158.82 ± 8.20 > 127.94 ± 20.03; (*p* < 0.001). CG > HEG = 125.50 ± 18.62 < 127.94 ± 20.03; (*p* = n.s.). BMI: T0 vs. T1 no differences in number of overweight (*p* = n.s.)
Pbert L. et al. [17] 2016; USA; RCT	n = 126 (EG: 58; CG: 68) Males: n = 42 (37.83%) Age: EG:16.5 ± 1.23; CG:16.3 ± 1.20 Enrollment period: September 2012–June 2013 Setting: High Schools	EG: Lookin’ Good Feelin’ Good. (i) School nurse-delivered counseling intervention; (ii) After-school exercise program. CG: No intervention. Time: (i) Phase of six weekly 30 min individual sessions followed by a maintenance phase of six monthly sessions and brief weekly weigh-ins; (ii) three sessions per week structured to increase exercise enjoyment. Duration: 8 months.	*Primary Outcomes:* Physical Activity Levels (number of days physically active in past 7 days); Sedentary Behavior (hours play video/computer games or watching TV on average school day in past 7 days); BMI; Dietary Intake *Methods/Questionnaire/Test:* ActiGraph Model GT1M (for a 7 day period); two items from the Youth Risk Behavior Survey; BMI (kg/m^2^); 24 h dietary recall interview *Other Outcomes:* ---	**T0 vs. T1 differences between EG and CG.** Physical Activity Levels (number days physically active in past 7 days): 0.89 (0.25–1.53); (*p* = 0.007). Physical Activity Levels (%Time spent in MVPA each day): −0.76 (−4.63–3.10); (*p* = n.s). **Sedentary Behavior:** −0.01 (−0.43–0.41); (*p* = n.s.). BMI: −0.14 (−1.09–0.81); (*p* = n.s.). Dietary Intake: Students in EG compared with CG schools reported eating breakfast on significantly more days/week. The other data on dietary intake are not statistically significant.
Choo J. et al. [20] 2020; South Korea; RCT	n = 104 (EG:49; CG:55) Males: n = 57 (54.8%) Age: EG:9.9 ± 1.18; CG:10.1 ± 1.27 Enrollment period: Recruitment in June 2017 Setting: Community Child Centers	EG: Multi-level interventions of child, parent, and center-level strategies. (i) Child-level: six weekly sessions for healthy eating and six weekly sessions for healthy activity; (ii) Parent-level: one session of group teaching, two home visits, three telephone counseling sessions, 12 weekly text messages; center-level: 12 sessions educational curriculum for heathy eating and activity and secured the physical environment for operating educational classes; the researcher educated the faculty members (directors, teachers, cooks) to be aware about obesity, to display posters regarding lifestyle, to adopt policies such as no sugar-sweetened. CG: No intervention. Time: No information. Duration: 12 weeks.	*Primary Outcomes:* Knowledge of Healthy Lifestyle Behaviors; Healthy Lifestyle Behavior; Obesity Status; BMI z score *Methods/Questionnaire/Test:* 18 item questionnaire developed by the PI (score 0–18); Healthy Lifestyle Behaviors = Self-Reported (score 0–9); BMI z score *Other Outcomes:* Parenting Behaviors	**T0 vs. T1 differences between EG and CG.** Knowledge of Healthy Lifestyle Behaviors: T0 EG 13.0 ± 1.74, T1 EG 14.8 ± 1.45; T0 CG 13.7 ± 2.04, T1 CG 14.2 ± 1.88; (*p* = 0.026). Healthy Lifestyle Behaviors: T0 EG 2.4 ± 1.56, T1 EG 3.4 ± 2.12; CG T0 3.3 ± 1.68, T1 CG 2.9 ± 1.62; (*p* < 0.01). Obesity Status: T0 EG 32.7%, T1 EG 36.7%; T0 CG 38.2%, T1 CG 41.8%; (*p* = n.s.). BMI z-score: T0 EG 0.8 ± 1.36, T1 EG 0.9 ± 1.36; T0 CG 1.3 ± 1.24, T1 CG 1.3 ± 1.22; (*p* = 0.05).

**Table 3:** Quality assessment of RCTs and observational studies.

Authors	Study Design	Tool for Assessment	Quality
Choo et al., 2020 [20]	RCT	Cochrane ROB2 Tool	Some concern
Pbert et al., 2016 [17]	RCT	Cochrane ROB Tool	Some concern
Robbins et al., 2019 [15]	RCT	Cochrane ROB Tool	High
Altunkurek et al., 2018 [21]	RCT	Cochrane ROB2 Tool	Some concern
Wright et al., 2013 [18]	RCT	Cochrane ROB2 Tool	Some concern
Ham et al., 2016 [19]	Quasi-experimental	ROBBINS-I	Moderate
Robbins et al., 2012 [16]	Quasi-experimental	ROBBINS-I	Low

## Data Availability

All data and materials are available upon written request to the corresponding author.
